# Supplementary material for: NF-κB Decoy Oligodeoxynucleotide-Loaded Poly Lactic-co-glycolic Acid Nanospheres Facilitate Socket Healing in Orthodontic Tooth Movement
Source: Int J Mol Sci. 2024 May 10;25(10):5223. doi: 10.3390/ijms25105223 (PMC11121581; doi:10.3390/ijms25105223)
Supplement: Supplementary file 1 [file ijms-25-05223-s001.zip › Figures.pdf]

Supplementary Information

Materials and Methods

Decoy ODN nuclear medicine

The concentration of ScD in the ScD and ScD-loaded PLGA (PLGA-ScD) solutions, as well as that of NfD in the NfD and NfD-loaded PLGA (PLGA-NfD) solutions, was 0.02% (*w/v*) (0.2 mg/mL). The concentration of hydroxypropyl cellulose-H was 3.3% (*w/v*) with 2.0% PLGA nanoparticles (20 mg/mL). The particle sizes of PLGA-ScD and PLGA-NfD were measured using the dynamic light scattering method, and the content percentage (%) of ODNs in the PLGA nanoparticles was measured using UV spectrophotometry. The encapsulation efficiency of ODN into the PLGA particles was calculated using the following formula: [active pharmaceutical ingredients (API) / API + PLGA + polyvinyl alcohol (PVA)].

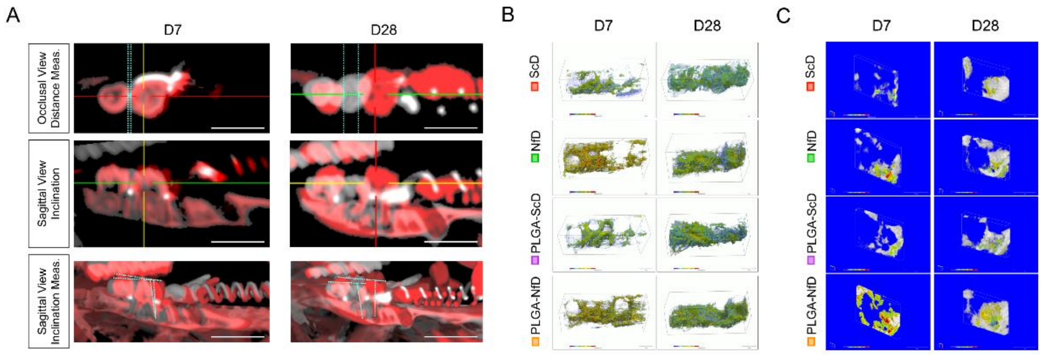

Figure S1. Micro-computed tomography (CT) analyses for OTM modality evaluation. **(A)** Micro-CT in superimposition mode for OTM modality evaluation. Micro-CT superimposition images acquired on D0 and D7 (gray), and D28 (red). Superimposition images from the sagittal and transverse views are shown to represent the amounts and directions of orthodontic tooth movement in the NfD group. Scale bar = 3 mm. **(B)(C)** Representative 3D video of the volume of interest (VOI) at M2 periodontium and M1 tooth-extraction socket (VOI1), and at the M2 mesial periodontium (VOI2). Trabecular 3D-bone mineral density (BMD)-generated VOI was used as measurement area for the alveolar bone analysis. VOI1 video and VOI2 images are shown. The BMD value is indicated by the BMD color transition scale. Scale bar = 1 mm. D7, post-extraction day 7; D28, post-extraction day 28; NfD, naked NF-κB decoy group; PLGA-NfD, NF-κB decoy ODN-loaded PLGA nanosphere group; PLGA-ScD, scrambled decoy ODN-loaded PLGA nanosphere group; ScD, naked scrambled decoy group.

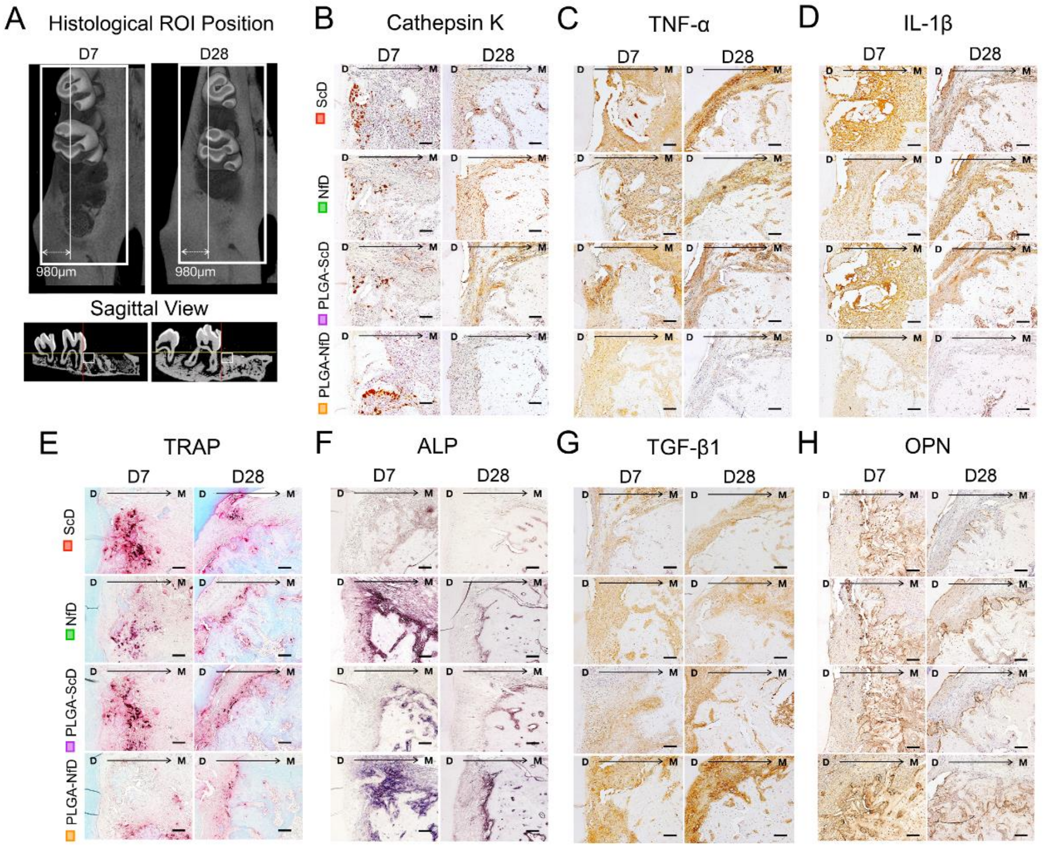

Figure S2. Histological analyses for OTM modality evaluation. **(A)** Analogous micro-computed tomography (CT) images for histological region of interest (ROI) position. Representative micro-CT images of D7 and D28 samples are shown, and the histological ROI position was determined from 980 μm of the buccal plane border in the volume of interest (VOI)1 of M2 OTM following M1 tooth-extraction. ROI was determined to be a region of total grid area of 0.5356742 mm^2 (1,311 × 409 μm) in the mesial side alveolus and periodontium of M2. Analysis was performed after obtaining three randomized tissue sections for each sample with four periodontal ligament tissue images at 200× magnification, as shown in the representative histological diagram.

Representative images of immunohistochemical staining of **(B)** cathepsin K, **(C)** TNF-α, **(D)** IL-1β, **(G)** TGF-β1, and **(H)** OPN, as well as **(E)** TRAP and **(F)** ALP staining, are shown. Representative image (original magnification: 100×) is shown for each histological assay. Black arrow indicates OTM direction. Scale bar = 100 μm. D, distal direction; D7, post-extraction day 7; D28, post-extraction day 28; M, mesial direction; NfD, naked NF-κB decoy group; PLGA-NfD, NF-κB decoy ODN-loaded PLGA nanosphere group; PLGA-ScD, scrambled decoy ODN-loaded PLGA nanosphere group; ScD, naked scrambled decoy group.
