# Supplementary material for: NF-κB Decoy Oligodeoxynucleotide-Loaded Poly Lactic-co-glycolic Acid Nanospheres Facilitate Socket Healing in Orthodontic Tooth Movement
Source: Int J Mol Sci. 2024 May 10;25(10):5223. doi: 10.3390/ijms25105223 (PMC11121581; doi:10.3390/ijms25105223)
Supplement: Supplementary file 1 [file ijms-25-05223-s001.zip › Table S1.pdf]

Key resource table

Key resources table				
**Reagent type (species) or resource**	**Designation**	**Source or reference**	**Identifiers**	**Additional information**
Strain, strain background (Rattus norvegicus) (male)	Wistar/ST	SLC, Japan	RRID:RGD_2314928	
Chemical compound, drug	Medetomidine	Nippon Zenyaku Kogyo Co., Ltd., Japan		
Chemical compound, drug	Midazolam	Teva Takeda Yakuhin Ltd., Japan		
Chemical compound, drug	Butorphanol	Meiji Seika Pharma Co., Ltd., Japan		
Other	Nickel-titanium closed coil tension spring	Tomy International, Tokyo, Japan	Catalog No. 509-29	
Other	Light-cured composite resin	GC, Tokyo, Japan	Catalog No.213345K75	

Commercial assay or kit	Paraformaldehyde	FUJIFILM Wako Pure Chemical Corporation, Japan	Product Code: 163-20145	
Commercial assay or kit	Disodium ethylenediamine tetraacetate (EDTA)	Nacalai Tesque, Inc., Japan	Product Code: 1511145	
Commercial assay or kit	TRAP/ALP stain kit	Fujifilm Wako Pure Chemical, Osaka, Japan	Product No: 294-67001	
Antibody	Rabbit Anti-TNF-alpha Polyclonal Antibody, Unconjugated	Bioss, Woburn, MA, USA	Bioss Cat# bs-2081R, RRID:AB_10856075	IHC dilution ratio: 1:400
Antibody	Rabbit Anti-IL-1 Beta/IL-1B Polyclonal Antibody, Unconjugated	Bioss, Woburn, MA, USA	Bioss Cat# bs-0812R, RRID:AB_10855142	IHC dilution ratio: 1:400
Antibody	Rabbit Anti-TGF Beta 1 Polyclonal Antibody, Unconjugated	Bioss, Woburn, MA, USA	Bioss Cat# bs-0086R, RRID:AB_10856457	IHC dilution ratio: 1:400

Antibody	Rabbit Anti-osteopontin Polyclonal Antibody, Unconjugated	Abcam, Cambridge, UK	Abcam Cat# ab8448, RRID:AB_306566	IHC dilution ratio: 1:400 IF dilution ratio: 1:200
Antibody	Rabbit Anti-cathepsin K Polyclonal Antibody, Unconjugated	Abcam, Cambridge, UK	Abcam Cat# ab19027, RRID:AB_2261274	IHC dilution ratio: 1:400 IF dilution ratio: 1:150
Antibody	Mouse Anti-NF-κB p65 Monoclonal (F-6) Antibody	Santa Cruz Biotechnology, Dallas, TX, USA	Santa Cruz Biotechnology Cat# sc-8008, RRID:AB_628017	IF dilution ratio: 1:150
Antibody	Rabbit Anti-RANKL Polyclonal Antibody, Unconjugated	Proteintech Group Inc., Rosemont, IL, USA	Proteintech Cat# 23408-1-AP, RRID:AB_2879273	IF dilution ratio: 1:150
Antibody	Rabbit Anti-Osteoprotegerin Polyclonal Antibody, Unconjugated	Abcam, Cambridge, UK	Abcam Cat# ab73400, RRID:AB_1640751	IF dilution ratio: 1:150

Antibody	Rabbit Anti-Osteocalcin Polyclonal Antibody, Unconjugated	Abcam, Cambridge, UK	Abcam Cat# ab93876, RRID:AB_10675660	IF dilution ratio: 1:200
Antibody	Donkey Anti-Rabbit IgG H&L (Alexa Fluor® 594)	Abcam, Cambridge, UK	Abcam Cat# ab150076, RRID:AB_2782993	Secondary Ab dilution ratio: 1:200
Antibody	Donkey Anti-Rabbit IgG H&L (Alexa Fluor® 488)	Abcam, Cambridge, UK	Abcam Cat# ab150073, RRID:AB_2636877	Secondary Ab dilution ratio: 1:200
Commercial assay or kit	Bovine Serum Albumin	Vector Laboratories, Inc., Burlingame, CA, USA	Vector Laboratories Cat# B-2007, RRID:AB_2336258	Dilution conc. 5%
Commercial assay or kit	Hydrogen peroxide	Abcam, Cambridge, UK	Product Code: ab64218	
Commercial assay or kit	VECTASTAIN Elite ABC Rabbit IgG Kit	Vector Laboratories, Inc., Burlingame, CA, USA	Product Code: PK-6101	
Commercial assay or kit	3,3′-Diaminobenzidin	Abcam, Cambridge, UK	Product Code: ab64238	

	e (DAB) substrate kit			
Commercial assay or kit	Hematoxylin	FUJIFILM Wako Pure Chemical Corporation, Japan	Product Code: 131-09665	
Commercial assay or kit	Vector® TrueVIEW® Autofluorescence Quenching Kit	Vector Laboratories, Inc., Burlingame, CA, USA	Vector Laboratories, # SP-8400	
Commercial assay or kit	VECTASHIELD® Vibrance™ Antifade Mounting Medium with DAPI	Vector Laboratories, Inc., Burlingame, CA, USA	Vector Laboratories, # H-1800-2	
Commercial assay or kit	TRIzol® reagent	Life Technologies Invitrogen; Thermo Fisher Scientific, Carlsbad, CA, USA	Product Code: 15596026	
Commercial assay or kit	PrimeScript™ RT Master Mix	Takara Bio, Otsu, Shiga, Japan	Product Code: RR036A	
Commercial assay or kit	Probe qPCR Mix	Takara Bio, Otsu, Shiga, Japan	Product Code: RR391A	
Sequence-based reagent	rat *Gapdh* (glyceraldehyde-	TaqMan Gene Expression Assay	Assay ID: Rn01775763_g1	

	3-phosphate dehydrogenase) mRNA	primers (Applied Biosystems; Thermo Fisher Scientific, Foster City, CA, USA)		
Sequence-based reagent	rat *Il1b* (interleukin 1 beta) mRNA	TaqMan Gene Expression Assay primers (Applied Biosystems; Thermo Fisher Scientific, Foster City, CA, USA)	Assay ID: Rn00580432_m1	
Sequence-based reagent	rat *tnf* (tumor necrosis factor) mRNA	TaqMan Gene Expression Assay primers (Applied Biosystems; Thermo Fisher Scientific, Foster City, CA, USA)	Assay ID: Rn01525859_g1	
Sequence-based reagent	rat *Tnfsf11* (receptor activation of nuclear factor-κB ligand; Tnfsf11) mRNA	TaqMan Gene Expression Assay primers (Applied Biosystems; Thermo Fisher	Assay ID: Rn00589289_m1	

		Scientific, Foster City, CA, USA)		
Sequence-based reagent	rat *Tnfrsf11b* (osteoprotegerin; Tnfrsf11b) mRNA	TaqMan Gene Expression Assay primers (Applied Biosystems; Thermo Fisher Scientific, Foster City, CA, USA)	Assay ID: Rn00563499_m1	
Sequence-based reagent	rat *Tgfb1* (transforming growth factor-beta 1) mRNA	TaqMan Gene Expression Assay primers (Applied Biosystems; Thermo Fisher Scientific, Foster City, CA, USA)	Assay ID: Rn00572010_m1	
Sequence-based reagent	rat *Spp1* (secreted phosphoprotein 1) mRNA	TaqMan Gene Expression Assay primers (Applied Biosystems; Thermo Fisher Scientific, Foster City, CA, USA)	Assay ID: Rn00681031_m1	

Sequence-based reagent	rat *Ctsk* (cathepsin K) mRNA	Takara Bio, Otsu, Shiga, Japan	Assay ID: NM_031560.2	
Sequence-based reagent	rat *Fgf2* (cathepsin K) mRNA	Takara Bio, Otsu, Shiga, Japan	Assay ID: NM_019305.2	
Sequence-based reagent	rat *Bglap* (bone gamma-carboxyglutamate protein) mRNA	TaqMan Gene Expression Assay primers (Applied Biosystems; Thermo Fisher Scientific, Foster City, CA, USA)	Assay ID: Rn00566386_g1	
Software, algorithm	TRI/3D-BON-FCS 3D trabecular bone analysis software	RATOC System Engineering Co., Tokyo, Japan		
Software, algorithm	ImageJ scientific software	ImageJ version 1.52; National Institutes of Health, Bethesda, MD, USA	RRID:SCR_003070	
Software, algorithm	NIS-Elements D Imaging	Nikon, Tokyo, Japan	RRID:SCR_014329	

	Software Version 2.30			
Software, algorithm	IBM SPSS Statistics for Windows, version 27.0	IBM Corp., Armonk, NY., USA	RRID:SCR_019096	
Software, algorithm	GraphPad Prism 9 (version 9.3.1)	GraphPad Software Inc, San Diego, CA, USA	RRID:SCR_002798	
